# Determinants and implementation strategies to implement a reflection method for guideline-based informal caregiving in community nursing in the Netherlands: a mixed-method study

**DOI:** 10.1136/bmjopen-2024-097103

**Published:** 2025-12-05

**Authors:** Nicole Vullings, Marjo Maas, Marian Adriaansen, Hester Vermeulen, Philip Van der Wees, Maud Heinen

**Affiliations:** 1 Radboud Institute of Health Sciences, Scientific Institute for Quality of Healthcare, Radboud University Medical Center, Nijmegen, the Netherlands; 2 Faculty of Nursing Studies, HAN University of Applied Sciences Institute of Nursing Studies, Nijmegen, the Netherlands; 3 Faculty of Allied Health Sciences, HAN University of Applied Sciences, Nijmegen, the Netherlands

**Keywords:** Implementation Science, Nursing Care, Quality Improvement, Protocols & guidelines, Community-Based Participatory Research

## Abstract

**Abstract:**

**Aim:**

This study aims to identify key determinants and strategies for effectively implementing a reflection method to support adequate use of the ‘Informal Care’ guideline within community nursing. The SPARK (Self & Peer Assessment to Reflect on Quality Standards) reflection method, developed in an earlier participatory design-based study, is a structured group reflection approach designed to help nurses and nursing assistants reflect on and apply guideline recommendations in daily practice.

**Design:**

A mixed method study.

**Setting:**

Six community care organisations in the Netherlands.

**Participants and interventions:**

This mixed-method study collected qualitative data through observations and video recordings of group meetings with community nurses, nursing assistants and a patient representative, alongside quantitative questionnaires. This project included design and test group meetings to develop and evaluate prototypes of the reflection method. Observations were discussed, and video recordings were thematically analysed. The Measurement Instrument for Determinants of Innovations questionnaire was used to identify key determinants for effective implementation. The questionnaire results were analysed descriptively, using the Tailored Implementation in Chronic Diseases (TICD) framework to present preliminary determinants for validation. Implementation strategies were then selected in a group meeting. Based on this input, the research group operationalised the selected implementation strategies.

**Results:**

Twenty-nine determinants for implementing the reflection method were identified across seven TICD domains, including barriers such as limited support, knowledge and time, and facilitators such as team collaboration and prevention of caregiver overload. Based on these findings, three implementation strategies, namely knowledge enhancement, coaching development and leadership strengthening, were formulated to support integration into community nursing practice.

**Conclusion:**

This study identified key determinants and strategies for implementing a reflection method in community nursing. While several determinants align with existing literature, context-specific determinants related to the heterogeneous group of registered nurses and certified nursing assistants also emerged. Strengthening guideline knowledge, coaching competencies and leadership is essential for sustainable, guideline-based reflection in practice.

**STRENGTHS AND LIMITATIONS OF THIS STUDY**

- Embedding the implementation meeting within the development process of the reflection method.
- Using multiple data sources (observations, video recordings and questionnaires).
- Using data from a 3.5 year project for identifying the determinants for good implementation of the reflection method.
- Inclusion of only one patient representative during the project.
- Limited number of respondents at the implementation meeting (n=9).

## Introduction

 The implementation of research findings into clinical practice remains a challenge, despite the large amount of evidence that has been developed in the field of implementation.^1 2^ While new care methods, guidelines and protocols are continuously being developed, many research findings are often never translated into policy and/or practice, resulting in little to no impact on actual care delivery. The success of a new intervention or guideline depends not only on its scientific foundation but also on how effectively they are implemented in day-to-day practice.^2 3^ Within the realm of healthcare implementation, the adoption of guidelines is particularly challenging. Guidelines are designed to facilitate evidence-based practice behaviour, with the aim of improving the quality of client care, reducing unwanted practice variations and promoting patient well-being.^4^,^6^ Clinical guidelines have proven effective in enhancing health outcomes and care processes.^7^ Despite the advantages of using guidelines in practice, their implementation is often complex.^8^ This complexity is also evident in nursing, where the practicalities of patient care demand adaptability and a clear understanding of guideline application.

The use of clinical guidelines in daily practice is challenging because nurses are often unaware of existing guidelines, and even when they are, they may find them too extensive and not fully applicable to individual patients.^9 10^ Furthermore, nurses frequently have limited time for quality-improvement activities, such as the implementation of guidelines.^4^,^6^ In community nursing, an additional challenge is the solitary nature of the profession, which further reduces the application of guideline recommendations and the use of related interventions and tools.^5 11^

To facilitate the use of guidelines by community nurses and certified nursing assistants, it is important to develop methods that support effective implementation. In the Netherlands, the national nursing guideline ‘Informal Care’ was developed to support community nurses and nursing assistants in identifying, supporting and collaborating with informal caregivers.^12^ The guideline provides evidence-based recommendations on how nurses can prevent and reduce caregiver burden and promote sustainable informal care.

To enhance the use of this guideline in daily practice, the SPARK reflection method was developed through a participatory design-based research approach.^13^ SPARK, an acronym for ‘Self & Peer Assessment to Reflect on Quality Standards’, is a structured group reflection method that enables nurses and nursing assistants to discuss real-life care situations compared with guideline recommendations. The method combines self-reflection, peer discussions and action planning to bridge the gap between guideline-based care and everyday nursing practice.

The role of informal caregivers has become increasingly important in the Netherlands to safeguard the accessibility and affordability of healthcare. It is essential that older adults remain at home for as long as possible. In community care, registered nurses and certified nursing assistants play a crucial role in identifying, preventing and reducing the overload of informal caregivers. The ‘Informal Care’ guideline was developed in the Netherlands, to support them in their role.

The SPARK reflection method is designed to ensure that the ‘Informal Care’ guideline is applied effectively in practice. However, simply publishing the reflection method will not ensure its adequate application in practice. Therefore, for community nurses and certified nursing assistants to work with the SPARK reflection method, it is crucial that its implementation is well supported and facilitated.

## Method

### Aim

The aim of this study is to identify key determinants and select strategies for the effective implementation of a reflection method for adequate use of the ‘Informal Care’ guideline in community nursing.

### Study design, setting and participants

This study employed a mixed-methods design, combining qualitative and quantitative data collection and analysis. The qualitative data were sampled from existing observations and video recordings of reflection meetings from earlier participatory design-based studies conducted between 2021 and 2024, in which the SPARK reflection method was developed, tested and refined.^13 14^ These materials were re-examined in the current study to identify determinants relevant to the implementation of the reflection method. The quantitative data for this study were newly collected and included responses to the Measurement Instrument for Determinants of Innovations (MIDI) questionnaire and input from participants during an in-person implementation meeting with registered nurses, certified nursing assistants and a patient representative, in which determinants for implementation were prioritised and tailored implementation strategies were discussed.^8 15^

The earlier development studies of the SPARK reflection method involved design and test groups consisting of registered nurses and certified nursing assistants from six different home care organisations in the Netherlands, as well as one patient representative. For the current implementation study, participants from the original design group and the patient representative were invited to take part. The recruitment procedures for the design and test groups are described in detail in a previous publication on the development of the reflection method.^13^

### Data collection

The data collection process involved three phases: (1) the months leading up to the implementation meeting, (2) during the preparation of the implementation meeting and (3) during the implementation meeting.

Table 1 shows an overview of the data collection, data analysis and outcomes of the three phases

**Table 1:** Overview of the data sampling, data analysis and outcomes per phase

Sampling stage	Who	Data collection	Data analysis	Outcome
Phase 1 Prior to the implementation meeting	Research team (NV, MH and MM)	Qualitative data: video recordings and observations (NV, MH and MM) of SPARK design and testing meetings.	Data summaries including all notes on content, location and process, an overview of preliminary determinants of effective implementation was developed, with consensus reached.	Preliminary determinants of effective implementation of the reflection method using the TICD classification.^6^
Phase 2 Prior to the implementation meeting—preparing for the implementation meeting	Participants of the design and test groups of the SPARK project: Registered community nurses and certified nursing assistants (n=27)	Quantitative data: online questionnaire based on the Measurement Instrument for Determinants of Innovations (MIDI) questionnaire to identify determinants.^15^	Descriptive statistics analysis of the questionnaire results. Defining determinants.	Overview of facilitators and barriers for implementation of the reflection method based on the MIDI questionnaire and categorised using the TICD classification to enhance refinement during the following process.^6^
Phase 3 During the implementation meeting	Participants of the design group: Registered community nurses, certified nursing assistants and a patient representative (n=9) Research team (NV, MH and MM).	Quantitative data: participants selected a top four of most important determinants from the overview of preliminary identified determinants.	Discussion about the preliminary determinants.	Top four of the most important determinants.
Phase 3 During the implementation meeting	Participants of the design group: Registered community nurses, certified nursing assistants and a patient representative (n=9) Research team (NV, MH and MM).	Qualitative data: participants were asked to select one determinant to develop a tailored strategy (work format named ‘World Café’).	Iterative process to arrive at fully described strategies.	Tailored strategies for three different determinants.

SPARK, Self & Peer Assessment to Reflect on Quality Standards; TICD, Tailored Implementation in Chronic Diseases.

#### Phase 1: data from the design and test meetings

In the first phase of data collection, conducted prior to the implementation meeting, the research team analysed existing video and observation data to identify determinants influencing the implementation of the reflection method. In this study, determinants were defined according to Fleuren *et al* as ‘factors that may facilitate or hinder the implementation of an innovation in healthcare practice’.^15^ The analysed data included video recordings and observations from all test and design meetings conducted during the development of the SPARK reflection method.

All video and observation data were analysed, and consensus was reached on the preliminary determinants for effective implementation of the SPARK reflection method. The list of determinants (barriers and facilitators) identified by three researchers (NV, MH and MM) from the video recordings and observations was categorised using the TICD classification (Tailored Implementation for Chronic Diseases).^6^

#### Phase 2: data collected during the preparation of the implementation meeting: questionnaire

Prior to the implementation meeting, participants (see table 1) completed an online questionnaire based on the MIDI to identify determinants 2 weeks before the implementation meeting.

The MIDI^15^ is a 29-item questionnaire designed to identify factors influencing the use of an intervention. It comprises four scales measuring determinants related to: the innovation, the user, the organisation and the sociopolitical environment. Response options range from 1 (totally disagree) to 5 (totally agree). The MIDI has demonstrated strong psychometric properties, including good internal consistency (Cronbach’s alpha values typically above 0.70 for the subscales) and confirmed content and construct validity across multiple implementation contexts. The instrument’s structure allows researchers to assess barriers and facilitators at different levels within an organisation, providing a comprehensive understanding of implementation determinants.

In accordance with the MIDI guidelines, modifications and selections were made to fit the current context and aim of this study. Items were reformulated with a focus on the reflection method as the innovation, and seven items from the original list were excluded, resulting in a final questionnaire of 22 items.

For the scoring approach, item means were calculated following Fleuren *et al*’s recommended procedure. Items for which at least 50% of respondents selected *agree* or *totally agree* were classified as facilitators, while those for which 50% or more selected *neutral*, *disagree* or *totally disagree* were classified as barriers. The results provided an overview of facilitators and barriers for the implementation of the reflection method, based on the MIDI questionnaire and categorised using the TICD classification to enhance refinement during the following process.^6^

#### Phase 3: data from the implementation meeting

The implementation live meeting was organised and moderated by the first author (NV) in Nijmegen in June 2023 and lasted 2 hours. The meeting was attended by three researchers (NV, MH and MM), the design group of the SPARK project, four community nurses and four certified nursing assistants from four different participating organisations, and one patient representative.^13^

Its purpose was to identify the most relevant determinants for implementing the SPARK reflection method and to collaboratively formulate implementation strategies.

During the meeting, the list of identified determinants was presented to all participants. The group was divided into three subgroups, each seated at a separate table, following the World Café working format.^16^ Within each subgroup, participants were first asked to create a top four of determinants they considered most important to address. Next, each subgroup selected one key determinant from their top four to develop a tailored implementation strategy.

Using the World Café format, all participants engaged in a structured, collaborative dialogue to design strategies aligned with each prioritised determinant. Each subgroup discussed potential implementation strategies for their chosen determinant and documented their ideas on a structured paper format specifying what, how, where and when the solution should be applied. After 20 min, the paper format was rotated to the next table, allowing each group to provide input on all determinants. This process was repeated over three rounds, enabling cross-pollination of ideas as participants carried insights, themes and questions into subsequent discussions.

Each subgroup was accompanied by a researcher to take notes and contribute to the discussions, ensuring that the content and reasoning behind each strategy were accurately captured. Photos of each group’s written output were taken to support later documentation and analysis by the research team. The outcome of this meeting was the development of three tailored implementation strategies, each corresponding to one prioritised determinant.

### Data analysis

#### Determinants

To arrive at an initial overview of determinants for implementation, the video recordings, observation notes of the design and test meetings and the outcomes of the MIDI questionnaire were used. In this study, triangulation occurred through the combination of qualitative and quantitative data.^17^

All video recordings and observations were independently analysed by three researchers of the research team (NV, MM and MH). From this analysis, a comprehensive summary was created, including all notes on content, location and process. This summary was then discussed by the researchers, who were also present at the design and test meetings. From this discussion, an overview of preliminary determinants was developed, with consensus reached among these three researchers. During this discussion, the identified determinants were jointly categorised according to the domains of the TICD classification.

The results of the MIDI questionnaire were descriptively analysed by one researcher (NV). Three researchers (NV, MM and MH) analysed the results of the MIDI questionnaire and determined which components were regarded as barriers or facilitators for implementing the reflection method. A cut-off value of 50% was used for this analysis (totally agree and agree/neutral/totally disagree and disagree). In this way, critical determinants were included to develop a targeted strategy.^15^ Subsequently, each identified determinant was classified according to the domains of the TICD framework.

#### Implementation strategies

After the implementation meeting, one researcher (NV) analysed the completed paper formats (World Café) with suggested implementation strategies, structured the data and wrote down the core set of implementation strategies in a table.

### Patient and public involvement

One patient representative was involved in the implementation meeting to present the patient perspective on the most important determinants of implementation and tailored implementation strategies. Apart from this, patients and members of the public were not directly involved in the design, conduct, reporting or dissemination of the study.

## Results

### Sociodemographic characteristics

All participants who were involved in the development of the reflection method (n=27) received the MIDI questionnaire, of which 19 participants (70%) completed the questionnaire. All participants from the design group invited to the implementation meeting attended the session (n=9), of which four registered community nurses, four certified nursing assistants and one patient representative. The patient representative was digitally present due to practical reasons. Three participants in the implementation meeting had also completed the MIDI questionnaire during the preparation phase of this meeting. Table 2 presents the background characteristics from the registered nurses and certified nursing assistants who filled in the MIDI questionnaire.

**Table 2:** Background characteristics of participants who completed the MIDI questionnaire and attended the implementation meeting

	N (%)	N (%)
	MIDI	Meeting
Total	19	8 (100%)
Gender		
Male	1 (5%)	1 (13%)
Female	18 (95%)	7 (87%)
Working experience		
<1 year	3 (16%)	1 (13%)
1–5 year	6 (32%)	2 (25%)
6–10 year	1 (5%)	0
>10 year	9 (47%)	5 (62%)
Educational level		
Community registered nurse	9 (47%)	4 (50%)
Registered nurse	3 (16%)	1 (13%)
Certified nursing assistant	7 (37%)	3 (37%)

* without the patient representative.

MIDI, Measurement Instrument for Determinants of Innovations.

### List of determinants

#### Phase 1: data from the design and test meetings

The determinants for implementation of the reflection method derived from the video recordings and observations (phase 1) are presented in table 3.

**Table 3:** Determinants for implementation of the reflection method using the TICD classification identified from the video recordings and observations

Domain	Determinant/factor	Barrier (−)/ facilitator (+)	Summary/example
Innovation *reflection method*	Availability of materials in electronic record	–	Reflection method not yet integrated into digital systems.
	Availability of materials recommended by guideline	+	Materials available and aligned with guideline content.
Individual professional factors *registered nurses and certified nursing assistants*	Recording informal care dialogues with informal caregivers	±	Seen as challenging but useful.
	Requesting permission from caregivers	–	Difficult to ask already burdened caregivers for consent.
	Role, skills and knowledge of coach	–	Coaching competencies sometimes insufficient.
	Understanding of guidelines	±	Knowledge about what a guideline is and how to apply it varies.
	Motivation to try innovations	±	Willingness differs across professionals.
Patient factors *clients and informal caregivers*	Cooperation of informal caregivers	±	Willingness to participate in recordings varies.
Professional interactions	Team cohesion and collaboration	+	SPARK method stimulates cooperation and feedback culture.
	Multilevel participation	+	Involvement of all educational levels promotes inclusiveness.
Incentives and resources	Time to meet as a team	–	Limited time to reflect and discuss care.
	Time for dialogue with caregivers	+	Time investment improves caregiver support.
	Preparation time	±	Valuable but time-consuming.
	Accessibility of materials	+	Game materials facilitate meetings.
	Replacement when staff leave	–	Continuity is challenged by staff turnover.
Capacity for organisational change	Organisational support or direction	–	Limited guidance from management on use of method.
Social, political and legal factors	Policy on indication in community nursing	–	Unclear how informal care fits within indication policy.
	Funding for reflection method	–	Lack of structural time and financial resources.
	National direction/professional association	+	Availability through national bodies supports uptake.

−, barrier; +, facilitator; SPARK, Self & Peer Assessment to Reflect on Quality Standards; TICD, Tailored Implementation in Chronic Diseases.

#### Phase 2: data collected during the preparation of the implementation meeting: questionnaire

Table 4 presents the outcomes of the MIDI questionnaire (phase 2).

**Table 4:** Outcomes of the MIDI questionnaire completed by registered nurses and certified nursing assistants

Number of participants (%) MIDI questions	Totally disagree	Disagree	Neutral	Agree	Totally agree
1. The reflection method provides all the information and materials necessary to work with it effectively.	0 (0%)	1 (5%)	4 (21%)	11 (58%)	3 (16%)
2. The reflection method is too complicated for me to use.	4 (21%)	11 (58%)	4 (21%)	0 (0%)	0 (0%)
3. The reflection method aligns well with how I am accustomed to working.	0 (0%)	3 (16%)	7 (37%)	8 (42%)	1 (5%)
4. I find the effects of using the reflection method to be clearly visible.	0 (0%)	3 (16%)	5 (26%)	10 (53%)	1 (5%)
5. I find the reflection method suitable for my clients.	0 (0%)	2 (11%)	5 (26%)	9 (47%)	3 (16%)
6. Using the reflection method supports me in following the Informal Caregivers guideline.	0 (0%)	1 (5%)	4 (21%)	12 (63%)	2 (11%)
7. I expect that by working with the reflection method, I can contribute to preventing or reducing informal caregiver overload.	0 (0%)	0 (0%)	3 (16%)	15 (79%)	1 (5%)
8. It is part of my role to use the reflection method.	0 (0%)	0 (0%)	6 (32%)	12 (63%)	1 (5%)
9. Clients will generally be satisfied if I work according to this reflection method.	0 (0%)	1 (5%)	7 (37%)	11 (58%)	0 (0%)
10. Clients will generally cooperate if I act in accordance with the reflection method.	0 (0%)	1 (5%)	9 (47%)	9 (47%)	0 (0%)
11. I can count on sufficient support from my colleagues if I need it while working with the reflection method.	0 (0%)	1 (5%)	9 (47%)	9 (47%)	0 (0%)
12. I have sufficient knowledge to use the reflection method.	1 (5%)	2 (11%)	4 (21%)	11 (58%)	1 (5%)
13. I am expected (by your colleagues or supervisors) to act in accordance with the reflection method.	1 (5%)	5 (26%)	10 (53%)	3 (16%)	0 (0%)
14. If I wanted to, I would be able to act according to the reflection method.	0 (0%)	1 (5%)	2 (11%)	13 (68%)	3 (16%)
15. Measures have been taken in my organisation to onboard new employees in applying the reflection method.	2 (11%)	12 (63%)	4 (21%)	1 (5%)	0 (0%)
16. There is enough staff in our organisation to operate according to the reflection method.	0 (0%)	5 (26%)	9 (47%)	5 (26%)	0 (0%)
17. Sufficient financial resources are available to operate according to the reflection method.	0 (0%)	5 (26%)	12 (63%)	2 (11%)	0 (0%)
18. Our organisation provides me with enough time to integrate the reflection method into my (daily) work.	0 (0%)	3 (16%)	10 (53%)	5 (26%)	1 (5%)
19. Our organisation provides me with sufficient materials and facilities to operate according to the reflection method.	0 (0%)	5 (26%)	8 (42%)	5 (26%)	1 (5%)
20. I have easy access to information about the use of the reflection method in my organisation.	1 (5%)	4 (21%)	9 (47%)	5 (26%)	0 (0%)
21. The activities of the reflection method align well with existing laws and regulations.	0 (0%)	0 (0%)	8 (42%)	11 (58%)	0 (0%)
22. I am pleased that this reflection method exists.	0 (0%)	0 (0%)	6 (32%)	13 (68%)	0 (0%)

MIDI, Measurement Instrument for Determinants of Innovations.

The results showed that registered nurses and certified nursing assistants felt that the reflection method supported them in using the Informal Caregiver guideline (item 6; 74%) and assisted in preventing or reducing informal caregiver overload (item 7; 84%). About half of the registered nurses and certified nursing assistants (item 16; 47%) responded neutrally regarding having enough staff to operate according to the reflection method, sufficient financial resources available (item 17; 63%), enough time to integrate the reflection method into daily work (item 18; 53%) and sufficient materials and facilities to operate according to the reflection method (item 19; 42%).

Following the descriptive analysis of the MIDI questionnaire and the subsequent discussion among the researchers, 10 determinants were identified as potentially having a substantial impact. Of these, nine determinants exert a positive influence (facilitators), while one determinant exerts a negative influence (barrier). The 10 determinants from the MIDI questionnaire are presented in table 5. These determinants are regarded as affecting the implementation of the reflection method.

**Table 5:** Determinants (barriers and facilitators) from the MIDI questionnaire

Domain (MIDI)	Determinant	Barrier (−)/facilitator (+)	Domain (TICD)
Innovation	Complexity of the reflection method	+	Innovation factors
Innovation	Completeness of the reflection method	+	Innovation factors
Innovation	Visibility of outcomes of the reflection method	+	Innovation factors
Innovation	Relevance for client in using the reflection method	+	Innovation factors
User	Personal benefit/drawbacks	+	Individual health professional factors
User	Outcome expectations that the reflection method makes a contribution for the informal caregiver	+	Individual health professional factors
User	Professional obligation in using the reflection method	+	Individual health professional factors
User	Client satisfaction in using the reflection method	+	Patient factors
User	Self-efficacy in using the reflection method	+	Individual health professional factors
User	Knowledge about the reflection method	+	Individual health professional factors
Organisation	Replacement when staff leave	–	Incentives and resources

+, positive influence (facilitator); −, negative influence (barrier); MIDI, Measurement Instrument for Determinants of Innovations; TICD, Tailored Implementation in Chronic Diseases.

Finally, the analyses of the video recordings, observations as well as the quantitative data from the MIDI-questionnaire led to a list of determinants (n=29) that were allocated to the seven TICD classification domains: innovation factors (n=5), individual health professional factors (n=9), patient factors (n=2), professional interactions (n=4), incentives and resources (n=5), capacity for organisational change (n=1) and social, political and legal factors (n=3).

#### Phase 3: data from the implementation meeting

To address the identified determinants, implementation strategies were selected. From the list of determinants derived from the analyses of the MIDI questionnaire and the qualitative data, participants at the implementation meeting (n=9) selected four determinants they considered most important for the implementation: (1) knowledge, (2) professional time, (3) support and/or direction from the organisation to use of the reflection method and (4) the role, skills and guideline knowledge of the coach.

### Implementation strategies

Based on the four determinants, overarching implementation strategies were formulated: (1) knowledge, (2) coaching and (3) leadership. Table 6 gives an overview of the selected implementation strategies for each of the three determinants for implementation of the reflection method. The implementation strategies are detailed as follows.

**Table 6:** Implementation strategies for implementing the reflection method to enhance adequate use of the guideline informal care in daily practice

TICD domain	Determinant	Strategy	How	Who
Innovation factors Reflection method Informal Caregiving	Knowledge	**Knowledge** Know what a guideline is Know what the content of this particular guideline is	Prior to the first meeting of the reflection method, explain guidelines in general with the link to the purpose of the reflection method, for example: 5 min video Assessment A meeting	Making a video or assessment: professional association or SPARK project team The meeting: the quality officer
		Coach must make the transfer between participants (practice) and the guideline (theory)	Training or e-learning for the coaches	SPARK project team
Individual health professional factors Registered nurses and certified nursing assistants	The role, skills and guideline knowledge of the coach.	**Coaching** Coach is aware of the content of the guideline. Understanding the aim and the content of the reflection method. Understanding obstacles encountered during the use of the reflection method. Adequate coach competencies.	For strategy a−d: Reading the summary of the guideline Training for the coaches about: the content of the reflection method. Communication skills and motivational skills. Intervision between the coaches	For strategy a−d: The coach should be from their own organisation. The coach can be a quality officer, team coach or a community nurse. This also makes them more involved on the floor and allows the community nurse to be a leading character. The professional association and the SPARK team develop the training for the coaches.
Incentives and resources and individual health professional factors	Professional time	Leadership Create awareness by the organisation and the nurses to: Have a dialogue with the health insurance that they may cover time spent in the reflection method for informal caregiving. Initiate dialogues with the healthcare organisations to implement various components (see how) for the SPARK training and the costs.	For strategy a+b: The importance of caring for informal caregivers (with evidence). A training: encouraging more utilisation of informal caregiving objectives and assessment tools in healthcare to underscore their significance. Knowledge about indications so that nurses know that there may be additional client time if they indicate accordingly. Framing the SPARK trainings as case discussions. A set amount of money for the team and for an individual for training.	For strategy a+b: The management or board, quality officer or director of the community care organisation with the nurses. Training for the nurses in using the measurement tools, aims and indications for informal caregivers: quality officer or registered nurse.
Capacity for organisational change	Support	**Leadership** Create leadership and lobby skills to create: Willingness of the organisation to spare time and finances. Engage the municipality and citizens in the importance of caring for informal caregivers.	Emphasising the importance of informal care: indicating on informal care, using measurement tools, having dialogues with management. Dialogues with the municipality about the importance of caring for informal caregivers.	A community nurse discussing within the organisation’s board. Board of Directors or the management of the district care organisation.

SPARK, Self & Peer Assessment to Reflect on Quality Standards; TICD, Tailored Implementation in Chronic Diseases.

#### Implementation strategies: knowledge

Increasing knowledge about (1) what a guideline is and about (2) the content of the specific Informal Care guideline contributes to the effective implementation of the reflection method for acting on guidelines in daily practice. To achieve this, the research and design group of the SPARK project developed a guideline self-assessment tool. The aim of this self-assessment is to encourage registered nurses and certified nursing assistants to become curious about guidelines in general and the content of the specific guideline, encouraging them to look and explore the guideline prior to the first reflection meeting. See online supplemental appendix 1 for the self-assessment.

#### Implementation strategies: coaching

To successfully implement the reflection method, a coach with specific skills in the following areas is essential: (1) knowledge of guidelines, (2) the application of communication and motivation techniques and (3) fostering connections between coaches.

To enhance a coach’s proficiency in these skills, it would be beneficial to develop a targeted training programme. This programme should follow an interactive format that alternates between theoretical instruction and role-playing based on real-world scenarios. Role-playing can contribute as an educational strategy by allowing coaches to experience situations firsthand, discuss potential barriers and build confidence in their role as a coach. Several studies showed that using role playing is an effective educational strategy to teach competencies and skills.^18 19^

Moreover, it is crucial that the training provides opportunities, both during and after the sessions, for coaches to connect and share experiences. This peer interaction would enable coaches to learn from one another.

#### Implementation strategies: leadership

To successfully implement the reflection method, strengthening leadership is essential. Registered nurses need to develop leadership competencies that enable them to engage with healthcare organisations, policymakers and health insurers to emphasise the importance of supporting informal caregivers and ensuring sufficient professional time for reflection.

Within this strategy, leadership involves creating awareness at both organisational and professional levels regarding the time investment required for reflection and informal care support. This includes initiating dialogue with health insurers about the possibility of reimbursing time spent on the reflection method for informal caregiving, and engaging with healthcare organisations to incorporate the SPARK training and related costs into their structural planning.

Leadership development can be supported through established national and international programmes designed to enhance nurses’ leadership skills.^20^,^24^

## Discussion

This study identified determinants and selected implementation strategies for the implementation of the reflection method. Twenty-nine determinants were identified within seven TICD domains. The four most important determinants according to the participants were as follows: (1) the connection between the Informal Care guideline and the reflection method, (2) professional time, (3) support and/or direction from the organisation for the use of the reflection method and (4) the role, skills and knowledge of the coach in the reflection method. Implementation strategies across these three domains were developed based on the four identified determinants: (1) knowledge, (2) coaching and (3) leadership.

In this study, contradictions were observed regarding the determinant of knowledge. For instance, participants indicated in the MIDI questionnaire that they possessed sufficient knowledge to effectively use the reflection method. However, during the test meetings, it became apparent that there was insufficient knowledge about guidelines in general and more specifically the Informal Care guideline. This contradiction may be explained by an overestimation of professionals’ knowledge of guidelines, possibly related to overestimation bias in self-assessment.^25^ Various studies showed that healthcare professionals tend to overestimate their knowledge and their application in daily practice. This overestimation can be attributed to several factors, such as a tendency towards overconfidence and reliance on subjective feelings of confidence rather than on evidence-based knowledge.^25^,^27^ Healthcare professionals often only become aware of their actual level of knowledge when others ask them questions.^28^ Registered nurses and certified nursing assistants are not aware that they lack knowledge about guidelines, as guidelines are unfamiliar to many of them.^13^ To address this knowledge gap, a self-assessment was developed. This self-assessment was intended to give participants a realistic view of their knowledge about the guideline, and to stimulate their interest in the guideline. By posing questions about guidelines in general and the content of the specific Informal Care guideline, this intervention aligns well with the precontemplation stage for behavioural change.^29 30^

Several determinants that were identified in this study, such as time, training and organisational support, are also well-known determinants in the literature that play an important role in implementing innovations in healthcare.^5 31^ One determinant that is rarely mentioned in literature is knowledge of guidelines in general. Many studies show that physicians and nurses have only limited knowledge of the content of specific guidelines they work with.^32^,^34^ However, these studies do not indicate that they also have limited knowledge about what a guideline is in general as demonstrated in this study. The Consolidated Framework for Implementation Research (CFIR) highlights that knowledge about the intervention or guideline is a critical determinant for successful implementation.

The results of the MIDI questionnaire indicated that registered nurses and certified nursing assistants reported feeling supported by the reflection method in adhering to the guideline recommendations. However, this does not automatically imply that they will apply these guideline recommendations in daily practice. Considering the stages of behaviour change, the registered nurses and certified nursing assistants who feel supported by the reflection method (74%) are in the preparation or action phase.^29^ By addressing these two phases during the implementation process, for instance, by regularly incorporating the reflection method throughout the year and discussing it in team meetings, it is possible to sustain the action phase and facilitate the integration of guideline recommendations into their daily work (maintenance phase).^35^

While the previous findings highlight the importance of individual readiness and behavioural change, sustainable implementation also depends on contextual and organisational factors that support these processes. The findings of this study are consistent with existing literature on guideline implementation in nursing. Fontaine *et al* demonstrated that multifaceted strategies such as education, reminders and feedback are most effective when tailored to the local context and user needs.^36^ Similarly, the strategies identified in this study, related to knowledge, coaching and leadership, reflect a context-specific and multifaceted approach, emphasising that successful implementation depends on the alignment between professional competencies and organisational conditions. Cassidy *et al* emphasised that successful implementation strongly depends on end-user engagement and the presence of local champions.^37^ This aligns with the findings of this study, which show that coaches play a key role in promoting the sustained use of the reflection method, highlighting the importance of peer support. Likewise, Spoon *et al* found that time constraints, organisational support and staff motivation are crucial factors influencing guideline implementation.^38^ These determinants also emerged in this study, particularly the need for sufficient time and organisational facilitation for reflection meetings. The SPARK reflection method addresses these well-known barriers in a structured and practice-oriented way. Finally, Wolbers *et al* demonstrated that structured reflection enhances nurses’ ability to apply guideline recommendations in practice.^39^ The SPARK method builds on this by embedding reflection as a systematic, team-based learning process that fosters professional dialogue and shared learning, essential elements for sustainable behavioural change in practice.

### Strengths and limitations of this study

One significant strength of this study lies in embedding the implementation meeting within the development process of the reflection method. By considering implementation early on, the method can be tailored to local contexts and benefit from dissemination through established networks.^40 41^ Furthermore, involving end-users in the implementation meeting facilitates early issue identification and ensures alignment with practical needs,^42^ ultimately facilitating implementation that is contextually appropriate. ^43 44^A limitation of the study lies in the limited sample size of respondents during the implementation meeting (n=9). The small sample size may constrain the generalisability of the identified determinants and implementation strategies to the broader population of nurses and caregivers. Nevertheless, it is noteworthy that determinants were not solely derived from this implementation meeting but also encompassed data collected from the SPARK project and the MIDI questionnaire involving approximately 50 participants from various community care organisations with diverse demographic characteristics.

Furthermore, the presence of only one patient representative during the implementation meeting is noteworthy. While the reflection method primarily serves as a tool for registered nurses and certified nursing assistants, the inclusion of informal caregivers in deliberations regarding the integration of the reflection method into daily care routines could have been beneficial. This consideration is crucial, as the dynamics of the professional—informal caregiver/client relationship serve as an important motivator for nurses in delivering high-quality care.^45 46^ Therefore, it would be beneficial for future design research to involve more patient representatives at specific stages of the project.

### Implication for practice and further research

For the implementation of the reflection method, it is crucial to employ implementation strategies to address the facilitators and barriers. It is therefore recommended to commence with the implementation strategies targeting the three main identified determinants. Prior to the deployment of the reflection method, it is important for the coaches of the reflection method to develop awareness regarding their coaching role and develop competencies to guide participants in the reflection process. For participants, it is essential to develop awareness of their guideline knowledge through a self-assessment, and the purpose of the reflection method in advance.

To successfully embed the reflection method within the healthcare organisation, registered nurses and certified nursing assistants need awareness of how they can allocate time to put the reflection method into daily practice by receiving training from the healthcare organisation. Additionally, it is crucial for healthcare organisations to invest in developing leadership and advocacy skills among their registered nurses and certified nursing assistants so that they can promote the importance of care for informal caregivers at the local, regional and national levels.

In subsequent research, it would be recommended to assess the effectiveness of the implementation by examining whether there is increased utilisation of the Informal Care guideline in digital client reports.

### Conclusion

This study identified 29 determinants for the implementation of the SPARK reflection method within community nursing. Many determinants, such as time, training and organisational support, align with existing literature. At the same time, the findings highlight that the heterogeneous group of registered nurses and certified nursing assistants requires context-specific strategies and demonstrates that implementation in community nursing cannot rely on generic approaches alone. Successful implementation depends on the alignment between individual competencies (guideline knowledge and coaching skills) and organisational conditions such as leadership and support. These insights contribute to a deeper understanding of how reflection-based learning can be embedded within diverse community nursing settings to promote sustainable guideline use and professional development.

## Supplementary material

10.1136/bmjopen-2024-097103 online supplemental file 1

## Data Availability

Data are available upon reasonable request. All data relevant to the study are included in the article or uploaded as supplementary information.
