# Supplementary material for: Determinants and implementation strategies to implement a reflection method for guideline-based informal caregiving in community nursing in the Netherlands: a mixed-method study
Source: BMJ Open. 2025 Dec 5;15(12):e097103. doi: 10.1136/bmjopen-2024-097103 (PMC12684198; doi:10.1136/bmjopen-2024-097103)
Supplement: online supplemental file 1 [file bmjopen-15-12-s001.pdf]

Appendix 1 Self-assessment SPARK

Self-assessment

This self-assessment consists of nine questions about guidelines in general and the Informal Care Guideline. To find the answers, you can refer to the Informal Care Guideline during the self-assessment: www.venvn.nl/media/u5oboc0o/richtlijn-mantelzorg-21-9-2021-2.pdf

The following questions pertain to the use of guidelines in general, not specifically the Informal Care Guideline, and **are based on fictitious case scenarios** without the use of any real individuals.

1. **What is a guideline? A guideline ...**

   a. Provides recommendations for practice.

   b. Is derived from a detailed protocol.

   c. Provides recommendations that cannot be deviated from.

   d. Provides recommendations that are legally established.

2. **When you make a decision, it is based on Evidence-Based Practice (EBP) in practice. What are the three pillars of EBP?**

   a. Scientific evidence, patient preferences, and personal expertise.

   b. Patient preferences, personal expertise, and costs.

   c. Costs, personal expertise, and agreements within the team.

   d. Scientific evidence, agreements with health insurers, and government policy.

3. **Which guidelines are you familiar with?**

   - Guideline for Detection and Prevention of Healthcare-Associated Infections
   - Guideline for Healthy Sleep and Care for Sleep Problems
   - Pressure Ulcer Guideline
   - Loneliness Guideline

- Guideline and Handbook on Avoidance of Care in Primary Care
- Informal Care Guideline
- Guideline on Changed Sexual Health
- Reporting Guideline
- Guideline for Medication Preparation and Administration
- Skin Irritation Guideline
- Delirium Guideline
- Infection Prevention Guideline
- Handbook for Communication in Dementia Care
- I am not familiar with any of these national guidelines.

The following questions pertain to the content of the Informal Care Guideline.

Sophie Jonkers, 55 years old, works as a legal assistant and has three children aged 22, 20, and 17. She is also a caregiver for her 83-year-old mother, who has required increasing care over the past two years due to reduced mobility and type 2 diabetes. Home care provides assistance twice a day with daily care tasks. Sophie has gradually taken on more responsibilities, such as laundry, shopping, and managing finances.

1. The Informal Care Guideline recommends the SOFA model to assist in dialogs with informal caregivers. You can use this model to address the various roles of informal caregiving. What do the letters in the SOFA model stand for?
   a. Togetherness, Openness, Flexibility, and Authenticity
   b. Collaborating, Supporting, Facilitating, and Coordinating
   c. Togetherness, Supporting, Facilitating, and Authenticity
   d. Collaborating, Openness, Flexibility, and Coordinating

2. You hear Sophie mention that she actually lost her mother a year ago and that she misses her. Over the past year, it's become quite a burden, but "this is how we do it in our family," she says. Which role within the SOFA model does the informal caregiver seem to adopt here?

   a. Caregiver

   b. Support-seeker

   c. Involved party

   d. Victim

   e. Experienced expert

3. You have a dialog with Sophie, the informal caregiver, and together you complete her ecogram. According to the guideline, what is the purpose of an ecogram for informal caregivers? Mapping out ...

   a. The personal and professional relationships of the caregiver

   b. The relationships that fall within the caregiver’s sphere of influence

   c. The quality, extent, and perceived support of the caregiver’s relationships

   d. The sustainability of the caregiver’s relationships

   e. The relationships that affect the caregiver’s capacity

Mia and Theo Janssen, both 86 years old. Theo suffered a stroke (CVA) a year ago. After rehabilitation, he is now back home, but his mobility has decreased, and he uses a walker. The aphasia resulting from the stroke makes communication difficult, leading to frustration. Mia, his wife, finds it challenging that Theo can’t always express what he wants. She has canceled most of her activities to care for Theo.

4. Which instrument, according to the Informal Care Guideline, is the most useful to get an indication of Mia’s burden?

   a. Perceived Pressure from Informal Care (EDIZ) assessment tool

   b. Positive Experiences Scale (PES)

c. Screening Instrument for Adolescents with a Chronically Ill Parent (SACZO)

d. Perceived Pressure from Informal Care – Plus (EDIZ+) assessment tool

5. You administer the assessment tool to Mia. After reviewing Mia's responses, it becomes clear that she is experiencing a higher-than-average burden. What is, according to the guideline, a logical next step?

   a. Map out the tasks and characteristics of informal caregiving

   b. Involve a case manager

   c. Engage the social network to lighten the load

   d. Organize respite care
