# Supplementary material for: Determinants and implementation strategies to implement a reflection method for guideline-based informal caregiving in community nursing in the Netherlands: a mixed-method study
Source: BMJ Open. 2025 Dec 5;15(12):e097103. doi: 10.1136/bmjopen-2024-097103 (PMC12684198; doi:10.1136/bmjopen-2024-097103)
Supplement: Supplementary file 2 [file bmjopen-2024-097103.reviewer_comments.pdf]

PEER REVIEW HISTORY

BMJ Open publishes all reviews undertaken for accepted manuscripts. Reviewers are asked to complete a checklist review form and are provided with free text boxes to elaborate on their assessment. These free text comments are reproduced below.

ARTICLE DETAILS

Title (Provisional)

Determinants and implementation strategies to implement a reflection method for guideline-based informal caregiving in community nursing in the Netherlands: a mixed method study

Authors

Vullings, Nicole; Maas, Marjo; Adriaansen, Marian; Vermeulen, Hester; Van der Wees, Philip; Heinen, Maud

---

VERSION 1 - REVIEW

**Reviewer**	**1**
**Name**	**Lancia, Loreto**
**Affiliation**	**University of L'Aquila**
**Date**	**09-Jul-2025**
**COI**	**None**

Dear Authors,

thank you for the opportunity to review your manuscript entitled "Determinants and implementation strategies to implement a reflection method for guideline-based informal caregiving in community nursing: a mixed-method study."

This is an important and timely topic, addressing a crucial gap in the implementation of guidelines to support informal caregivers in community nursing. Your participatory and mixed-methods approach is highly relevant. However, several points require further clarification and revision to strengthen the methodological rigor and overall clarity of the manuscript.

MAJOR COMMENTS

• The introduction lacks a clear and concise description of the SPARK method and the "Informal Care" guideline. Please clearly define these elements early in the introduction to ensure readers unfamiliar with the Dutch context can fully understand the relevance and scope.

• The concept of "determinants" is central to the study but is not clearly defined or operationalized in the manuscript. Please provide a precise definition and explain how these determinants were identified and analyzed using the TICD framework.

• The term "implementation meeting" is mentioned multiple times before it is fully explained. Please clarify what this meeting entailed, who participated, what activities were conducted, and its specific purpose in the study timeline.

• The manuscript does not provide sufficient detail on inclusion/exclusion criteria, recruitment procedures, or how participants were allocated to different phases and activities (e.g., questionnaire, World Café discussions). Clarifying these aspects is crucial for reproducibility.

• The methods section should more explicitly describe what was recorded and observed, the duration of meetings, settings, and whether a topic guide was used during discussions.

• Clarify which components are qualitative and which are quantitative, and provide further detail on the psychometric properties and scoring approach of the MIDI questionnaire.

• Consider adding an introductory paragraph to the Results section summarizing the phases and data sources before detailing findings.

• Tables, especially Table 3, could be reformulated to improve conciseness and readability.

• The discrepancy between participants' self-reported knowledge (via MIDI) and actual observed knowledge gaps should be discussed more deeply, potentially referencing known overestimation biases in self-assessment literature.

MINOR COMMENTS

• The abstract would benefit from a brief explanation of the SPARK method and a clearer formulation of the conclusion.

• Clarify the distinction between data collected in this specific study versus prior SPARK project phases.

• Consider expanding references to similar reflective or guideline-implementation studies in community nursing to strengthen the discussion.

**Reviewer**	**2**
**Name**	**Ali, Salamat**
**Affiliation**	**Quaid-i- Azam University, Islamabad , Department of Pharmacy,**
**Date**	**25-Sep-2025**
**COI**	**None**

This is an excellent work and appreciate the contribution of authors to the literature. However, there are few suggestions from myside; 1. The write up needs to be revised for language check. 2. Conclusion also require minor revision in terms of quality words. 3. There are few old references and it is advised to include/ cite latest research work.articles

---

VERSION 1 - AUTHOR RESPONSE

**Reviewer 1**	**Author's response**	**Line**
The introduction lacks a clear and concise description of the SPARK method and the "Informal Care" guideline. Please clearly define these elements early in the introduction to ensure readers unfamiliar with the Dutch context can fully understand the relevance and scope.	The introduction was revised to include clear and concise information about the Informal Care guideline and the SPARK reflection method, ensuring that readers unfamiliar with the Dutch context can understand the background and relevance.	3-6<br><br>59-70
The concept of "determinants" is central to the study but is not clearly defined or operationalized in the manuscript. Please provide a precise definition and explain how these determinants were identified and analyzed using the TICD framework.	In response to the reviewer's feedback, the methods section was reviewed and expanded to include a literature-based definition of the concept of determinants, tailored to this study. Furthermore, the process of analyzing these determinants using the TICD framework has been explicitly described.	110-121
The term "implementation meeting" is mentioned multiple times before it is fully explained. Please clarify what this meeting entailed, who participated, what activities were conducted, and its specific purpose in the study timeline.	The term 'implementation meeting' is now clearly introduced at the beginning of the manuscript so that readers can understand its meaning and purpose from the outset.	92-96<br><br>143-149
The manuscript does not provide sufficient detail on inclusion/exclusion criteria, recruitment procedures, or how participants were allocated to different phases and activities (e.g., questionnaire, World Café discussions). Clarifying these aspects is crucial for reproducibility.	A more detailed description of the inclusion and exclusion criteria has been provided, clarifying how these relate to previous SPARK studies.	87-102
The methods section should more explicitly describe what was recorded and observed, the duration of meetings, settings, and whether a topic guide was used during discussions.	The methods section now provides a more transparent overview of which data were used, at what stage, and in which setting.	87-102

Clarify which components are qualitative and which are quantitative, and provide further detail on the psychometric properties and scoring approach of the MIDI questionnaire.	At the beginning of the methods section, a clearer distinction is now made between qualitative and quantitative data, including relevant information about their psychometric properties.	87-102<br><br>Table 1<br><br>123-136
Consider adding an introductory paragraph to the Results section summarizing the phases and data sources before detailing findings.	We considered adding an overview of the study phases in the results section but decided against it. Instead, we reorganized the methods section to improve transparency and provide readers with a clearer understanding of the research process.	Table 1<br><br>87-166
Tables, especially Table 3, could be reformulated to improve conciseness and readability.	Tables 1, 3, 5, and 6 were revised to enhance clarity, conciseness, and overall readability.	Table 1, 3,5 and 6
The discrepancy between participants' self-reported knowledge (via MIDI) and actual observed knowledge gaps should be discussed more deeply, potentially referencing known overestimation biases in self-assessment literature.	The discussion section now elaborates more thoroughly on the potential overestimation bias observed in the findings, supported by relevant literature.	323-329
The abstract would benefit from a brief explanation of the SPARK method and a clearer formulation of the conclusion.	In the abstract, an additional explanation of the SPARK method was included in the objective statement to provide better context for readers.	3-6
Clarify the distinction between data collected in this specific study versus prior SPARK project phases.	Throughout the methods section, we now more clearly distinguish between data derived from earlier project phases and data collected specifically for this study.	87-102<br><br>104-106<br>Table 1
Consider expanding references to similar reflective or guideline-implementation studies in community nursing to strengthen the discussion.	Finally, in the discussion section, a broader perspective on guideline implementation within nursing has been added, recognizing the limited amount of research conducted specifically within community nursing.	355-374

Reviewer 2	Author's response	Line
The write up needs to be revised for language check.	The entire manuscript has undergone a thorough language revision to improve clarity, consistency, and academic tone.	Entire manuscript
Conclusion also require minor revision in terms of quality words	The conclusion in both the abstract and the main manuscript has been revised and refined to enhance precision, coherence, and alignment with the study's findings.	22-26<br>414-422
There are few old references and it is advised to include/ cite latest research work.articles	All references throughout the manuscript have been reviewed, and several recent and relevant sources have been added to strengthen the scientific foundation of the paper.	Entire manuscript

VERSION 2 - REVIEW

Reviewer 1

Name Lancia, Loreto

Affiliation University of L'Aquila

Date 03-Nov-2025

COI

Dear Authors,

Thank you for your thorough revision and detailed responses.

I appreciate the substantial improvements made in clarity, methodological transparency, and overall structure. The key issues raised in the previous review have been well addressed.

In my opinion, the manuscript now reads coherently and meets the expected scientific standards.
